# Comparing CLIF-C ACLF, CLIF-C ACLF_lactate_, and CLIF-C ACLF-D Prognostic Scores in Acute-on-Chronic Liver Failure Patients by a Single-Center ICU Experience

**DOI:** 10.3390/jpm11020079

**Published:** 2021-01-29

**Authors:** Chao-Cheng Kuo, Chien-Hao Huang, Ching Chang, Pin-Cheng Chen, Bo-Huan Chen, Wei-Ting Chen, Yu-Pin Ho

**Affiliations:** 1 Division of Hepatology, Department of Gastroenterology and Hepatology, Chang-Gung Memorial Hospital, Linkou Medical Center, Taoyuan 333423, Taiwan; muscle210061@hotmail.com (C.-C.K.); o78714@gmail.com (C.C.); pin_chang0730@hotmail.com (P.-C.C.); spring03258@gmail.com (B.-H.C.); weiting1972@gmail.com (W.-T.C.); hoyupin@cgmh.org.tw (Y.-P.H.); 2 College of Medicine, Chang-Gung University, Taoyuan 333423, Taiwan

**Keywords:** liver cirrhosis, acute-on-chronic liver failure (ACLF), CLIF-C ACLF, lactate-adjusted CLIF-C ACLFs (CLIF-C ACLF_lactate_ score), CLIF-C ACLF-D, intensive care unit, area under the ROC curve (AUROC), error bars

## Abstract

Patients with liver cirrhosis have a higher risk of developing acute-on-chronic liver failure (ACLF). Poor prognosis with a high rate of short-term mortality leads to limited opportunities for further liver transplantation. Thus, precise prognostic evaluation of patients with ACLF is necessary before transplant surgery. In this study, a total of one hundred and thirty-five patients with ACLF admitted to the hepato-gastroenterologic intensive care unit (ICU) for intensive monitoring and treatment at Chang-Gung Memorial Hospital (CGMH, Linkou, Taiwan) were screened from November 2012 to April 2015 and tracked until April 2017. Three new prognostic scores of ACLF, including CLIF-C ACLF (Chronic Liver Failure Consortium Acute-on-chronic Liver Failure score), CLIF-C ACLF-D (CLIF-C ACLF Development score), and CLLF-C ACLF_lactate_ (lactate-adjusted CLIF-C ACLF score) were compared. The primary outcome considered was overall mortality. Mortality predictions at 28, 90, 180, and 365 days were also calculated. By area under the receiver operating characteristic curve (AUROC) analysis, the CLIF-C ACLF and CLIF-C ACLF-D scores were superior to CLIF-C ACLF_lactate_ scores in predicting 28-day mortality. The CLIF-C ACLF-D score had the highest AUROC in predicting overall mortality as well as at 90, 180, and 365 days. In conclusion, our study demonstrates that CLIF-C ACLF and CLIF-C ACLF-D scores are significant predictors of outcome in critical patients with liver cirrhosis and ACLF. The CLIF-C ACLF-D score may have a superior predictive power for the prediction of 3-month, 6-month, and one-year mortality.

## 1. Introduction

Patients with liver cirrhosis are more inclined to suffer acute decompensation complicated with organ failure, which is defined as having acute-on-chronic liver failure (ACLF) [1]. The syndrome usually requires admission to an intensive care unit (ICU) due to high mortality. Mortality rates among these patients treated in an ICU were over 50% and were 30–40% by day 28. Moreover, poor prognosis leads to limited chances for liver transplantation [2,3]. 

There are several features of ACLF that pose greater challenges for acute management and prognoses, such as increased susceptibility to infection [4,5] and complications associated with portal hypertension including variceal bleeding, refractory ascites, hepatic hydrothorax, hepatic encephalopathy, and hepatorenal syndrome [6,7]. The CANONIC (chronic liver failure (CLIF) Acute-on-Chronic Liver Failure in Cirrhosis) study [8] analyzed data from patients with cirrhosis and acute decompensation (AD) to establish the diagnostic criteria for ACLF. Based on CLIF-C OF (CLIF-C oragn failure) scores, the CLIF consortium created an acute-on-chronic liver failure score (CLIF-C ACLFs) and validated it ACLF patients hospitalized in an ICU [9]. The lactate-adjusted CLIF-C ACLF was recently developed (CLIF-C ACLF_lactate_ score) based on a multinational study in Germany and Austria, which functioned significantly better than the original CLIF-C ACLF in predicting 28-day mortality [10]. Furthermore, the up-to-date CLIF-C ACLF-D score was developed in the PREDICT (PREDICTing Acute-on-Chronic Liver Failure) study [11], which investigated the pathophysiological mechanisms of acute decompensation in liver cirrhosis. This new score was developed to predict the probability of a patient with AD developing ACLF within 3 months of hospital admission and showed a similar accuracy but smaller difference than other scores in the validation set [10].

A review of the literature shows minimal evidence comparing the two new prediction models (CLIF-C ACLF_lactate_ and CLIF-C ACLF-D) to the original CLIF-C ACLF score in predicting mortality among patients with ACLF. Furthermore, the major etiologies of cirrhosis in previous studies were alcohol and HCV (Hepatitis C Virus) [10,11]. In contrast, HBV (Hepatitis B Virus) infection is the most common cause of chronic liver disease in Eastern Asia [12]. The CLIF-C ACLF score was found to be statistically superior to the CTP (Child-Turcotte-Pugh) and MELD (Model for End-Stage Liver Disease) scores in predicting 28-day and overall mortality within an HBV-prevalent ACLF cohort in Taiwan [13]. In addition, sociocultural beliefs do not support procuring organs from deceased donors in traditional Asian societies. Therefore, donor rates are frequently lower than those in Western countries. Indeed, better utilization of living donor livers, avoiding unfruitful transplantation, and improving candidate selection are areas that desire improvements in Taiwan [14,15,16,17,18]. To address these needs, the aim of this study is to appraise the prognostic performance and clinical reliability of three scores for ACLF patients admitted to the ICU in a medical center in northern Taiwan.

## 2. Patients and Methods

### 2.1. ACLF Definition and Diagnosis

The European Association for the Study of the Liver (EASL)-CLIF consortium diagnostic criteria were adopted by us for the diagnosis of ACLF [8]. ACLF severity was based on EASL-CLIF consortium criteria [8,9]. In short, grade I ACLF was defined as having acute renal failure or any single organ failure associated with a serum creatinine level ranging from 1.5 to 1.9 mg/dL and/or hepatic encephalopathy grades 1 or 2; grade II was defined as having two organ failures; and grade III was defined as having three or more organ failure. 

### 2.2. Patient Selection

Patients who met the inclusion criteria comprise recurrent cirrhosis patients with ACLF admitted to our hospital’s hepato-gastroenterology ICU for intensive and specialized care from November 2012 to April 2015. Exclusion criteria included previously diagnosed HCC (hepatocellular carcinoma) before enrollment, age < 18 years old, pregnancy, ACLF score of 0 (absence of ACLF), orthotopic liver transplantation during follow-up, no available staging information during follow-up, and no available serum lactate level within the first 24 h after ICU admission.

### 2.3. Definition of Liver Cirrhosis and Decompensated Cirrhosis

The presence of liver cirrhosis was diagnosed based on a combination of characteristic clinical (e.g., jaundice, ascites, caput medusae, palmar erythema, spider angiomata, etc.), laboratory, and radiological findings (inhomogeneous hepatic texture or surface, rarefied hepatic central vein, an enlarged caudate lobe, splenomegaly or collateral veins, etc. in ultrasonography or computed tomography scanning) or via histology [6,7,19,20]. Clinical presentation of patients with decompensated cirrhosis is characterized by the presence of dramatic and life-threatening complications such as variceal bleeding, refractory ascites, spontaneous bacterial peritonitis, or hepatic encephalopathy [6,7].

### 2.4. Data Source and Collection

Data were collected from the hepato-gastroenterology ICU in Chang-Gung Memorial Hospital (CGMH), Linkou. Patient demographics, etiology of cirrhosis, peripheral complete blood count and differential count, serum biochemistry tests, oxygenation support, mechanical ventilator setting, Glasgow coma scale (GCS) score, vital signs (body temperature, blood pressure, and heart rate), urine output, and survival time were acquired from medical records. All scores and data were calculated according to their own formula, and data acquired at or within the 24 h of the first ICU admittance were utilized.

#### 2.4.1. Prognostic Score Computation

The CLIF-C ACLF score was calculated by combination of CLIF-C OF (CLIF-C organ failure) score, WBC (white blood cell) count, and age using the following formula: CLIF-C ACLF = 10 × (0.33 × CLIF-OFs + 0.04 × Age + 0.63 × ln(WBC count) − 2 [9]. The lactate-adjusted CLIF-C ACLF (CLIF-C ACLF_lactate_ score) was calculated using a combination of the CLIF-C ACLF score and serum lactate level using the following formula: CLIF-C ACLF_lactate_ = CLIF-C ACLFs + 8 × ln (lactate) − 7 [10]. The CLIF-C ACLF-D score was calculated using a combination of age, presence of ascites, WBC count, serum albumin, serum bilirubin, and serum creatinine using the following formula: CLIF-C ACLF-D score = ((0.03 × Age) + (0.45 × Ascites) + (0.26 × ln(WBC)) − (0.37 × Albumin) + (0.57 × ln(Bilirubin)) + (1.72 × ln(Creatinine)) + 3 × 10 [11].

#### 2.4.2. Primary Outcome and Scheduled Follow-Up Periods

The primary outcome was overall mortality. Mortality rates at 28, 90, 180, and 365 days were also enumerated as secondary outcomes. The conditions of the patients after hospital discharge were verified by telephone interview and/or analysis of chart records.

### 2.5. Statistical Analysis

The discrete variables are summarized as counts (percentages), and the continuous variables are summarized as mean ± SD. Nonnormally distributed variables are summarized as medians (IQR, interquartile range) and were log transformed for some statistical analyses as well as for graphical comparison. The categorical variables were expressed as frequencies and percentages and were compared with the Chi-square test. If more than 20% of the statistic cells showed an expected frequency less than 5, Chi-square test was replaced by Fisher’s exact test. Continuous variables between 28-day survivors and non-survivors were compared by Mann–Whitney U test. The area under the receiver operating characteristic (AUROC) curve was calculated to assess the accuracy of the CLIF-C ACLF, CLIF-C ACLF_lactate_, and CLIFACLF-D score in predicting survival. The capability of each score to predict mortality was then compared according to several scheduled follow-up periods by the Hanley and McNeil test. All statistical analyses were performed by IBM SPSS Statistics 26, and the figures were plotted by MedCalc statistical software v19.4.1. A *p*-value less than 0.05 was statistically significant.

## 3. Results

### 3.1. Demographic Characteristics of Critically Ill Patients with Cirrhosis and ACLF Admitted to ICU

**Figure 1:**
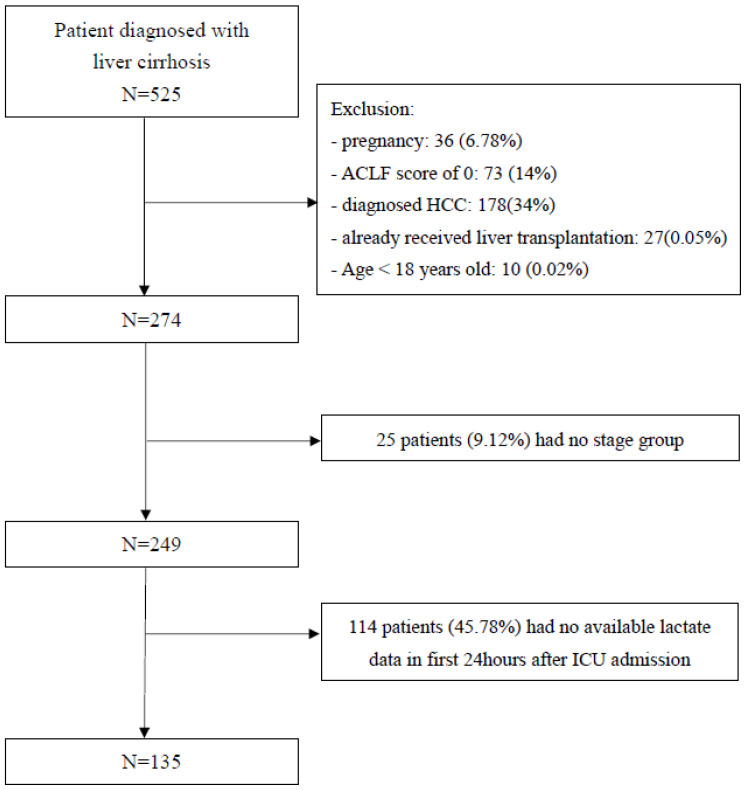
Flow chart demonstrating the process for patient recruitment, inclusion, and exclusion of ACLF cases in the intensive care unit.

From November 2012 to April 2015, a total of one hundred and thirty-five ACLF patients hospitalized to the hepato-gastroenterologic ICU were recruited after evaluating the inclusion and exclusion criteria (Figure 1) and were tracked until April 2017. The mean follow-up time was 427.54 days, and 95 patients died overall (70.37%). The patients were divided into two groups according to the survival status at day 28, and their baseline clinical parameters are shown in Table 1. The overall average age was 54.76 ± 14.06-year-old, and 100 patients (66.66%) were male. Male gender accounted for a greater proportion of survivors than non-survivors although males also made up the majority of the total population followed. For the etiology of cirrhosis, 40% had HBV, 16.29% had HCV, and 4.44% were HBV + HCV co-infected while 25.93% were alcoholics, 5.93% were alcoholics with HCV, and 7.41% had other etiologies including NASH (nonalcoholic steatohepatitis) or autoimmune hepatitis. The proportion of patients with grade I/II hepatic encephalopathy and their ages were similar between the two groups, whereas the proportion of patients with grade III/IV hepatic encephalopathy, serum bilirubin levels (14.1 mg/dL vs. 2.8 mg/dL, *p* < 0.001), INR (international normalized ratio) (2.1 vs. 1.5, *p* < 0.001), serum creatinine (1.63 mg/dL vs. 1.0 mg/dL, *p* < 0.001), and white blood cell count (10.4 × 10^9^/L vs. 8.1 × 10^9^/L, *p* = 0.013) were significantly higher in the non-survivors at 28 days. The serum albumin, serum sodium levels, and arterial PH were not significantly different between survivors and non-survivors. In terms of ACLF severity assessment, patients in the survivor group were more often classified as ACLF grade 1, while patients in the non-survivor group were more often classified as ACLF grade 3. For the precipitating events of ACLF, gastrointestinal hemorrhage (43%) accounted for the most proportion, followed by sepsis (22%), active alcoholism within the past three months before ICU admission (20%), and others (15%).

### 3.2. Values at 28, 90, 180, and 365 day Follow-Up among Survivors versus Non-Survivors

**Figure 2:**
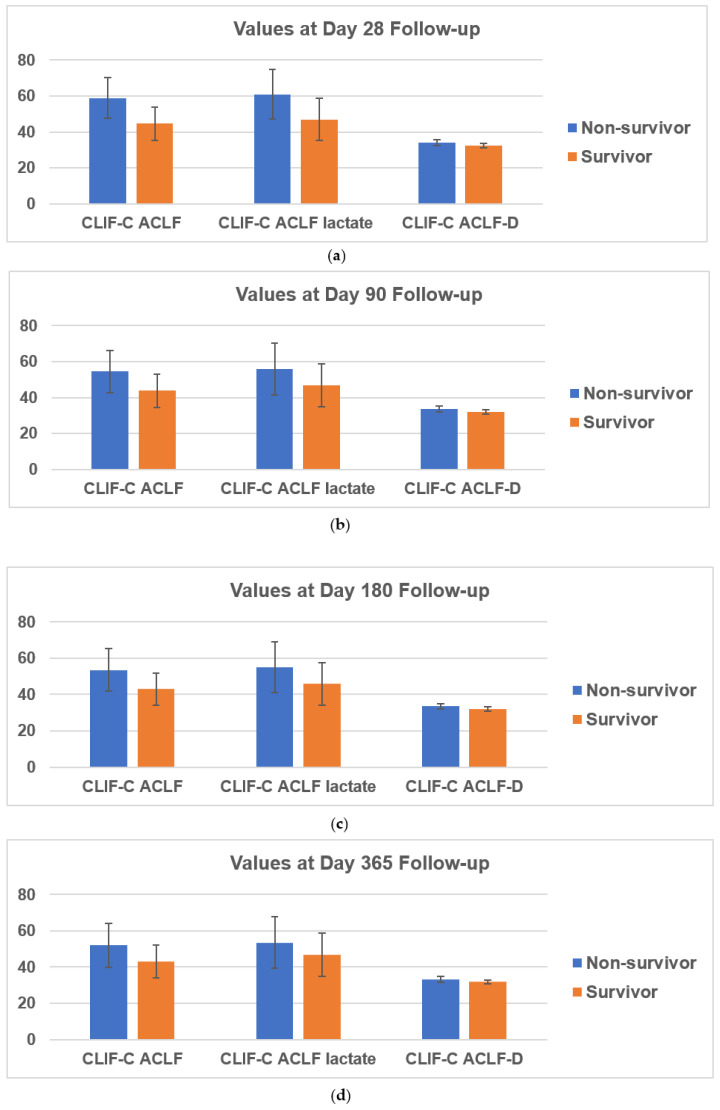
Error bar analysis with confidence intervals at 28-day (**a**), 90-day (**b**), 180-day (**c**), and 365-day (**d**) follow-up for each score compared between survivors and non-survivors.

Patients were divided into non-survivor (37 patients, 27.41%) and survivor (98 patient, 72.59%) based on mortality by day 28. Error bars represent one standard error with 95% confidence interval. For the values at day 28 follow-up, three prognostic scores were markedly increased in the non-survivor group (Figure 2a). The CLIF-C ACLF score, CLIF-C ACLF_lactate_ score, and CLIF-C ACLF-D score were 58.85 ± 11.40, 60.88 ± 13.71, and 34.03 ± 1.57 in the non-survivor group, which were significantly higher than that in the survivor group (44.55 ± 9.14, 46.91 ± 11.66, and 32.29 ± 1.17 respectively, all *p*-values < 0.01). Furthermore, we analyzed follow-up at 90, 180, and 365 days, also finding significantly higher scores in the non-survivor group than in the survivor group (Figure 2b–d). At 90 days, the CLIF-C ACLF score, CLIF-C ACLF_lactate_ score, and CLIF-C ACLF-D scores between non-survivors and survivors were 54.39 ± 11.83 vs. 43.73 ± 9.18, 55.75 ± 14.33 vs. 46.73 ± 11.83, and 33.61 ± 1.50 vs. 32.09 ± 1.12, respectively (all *p*-values < 0.001). At 180 days, scores between non-survivors and survivors were 53.44 ± 11.77 vs. 42.95 ± 8.81, 55.12 ± 14.00 vs. 45.88 ± 11.68, and 33.43 ± 1.54 vs. 32.03 ± 1.07, respectively (all *p*-values < 0.001). At 365 days, scores between non-survivors and survivors were 51.95 ± 11.96 vs. 43.25 ± 9.10, 53.45 ± 14.21 vs. 46.67 ± 11.93, and 33.29 ± 1.55 vs. 31.99 ± 1.03, respectively (*p* < 0.01, *p* = 0.003, *p* < 0.01).

### 3.3. AUROC Comparisons of Overall Mortality Prediction at Days 28, 90, 180, and 365

**Figure 3:**
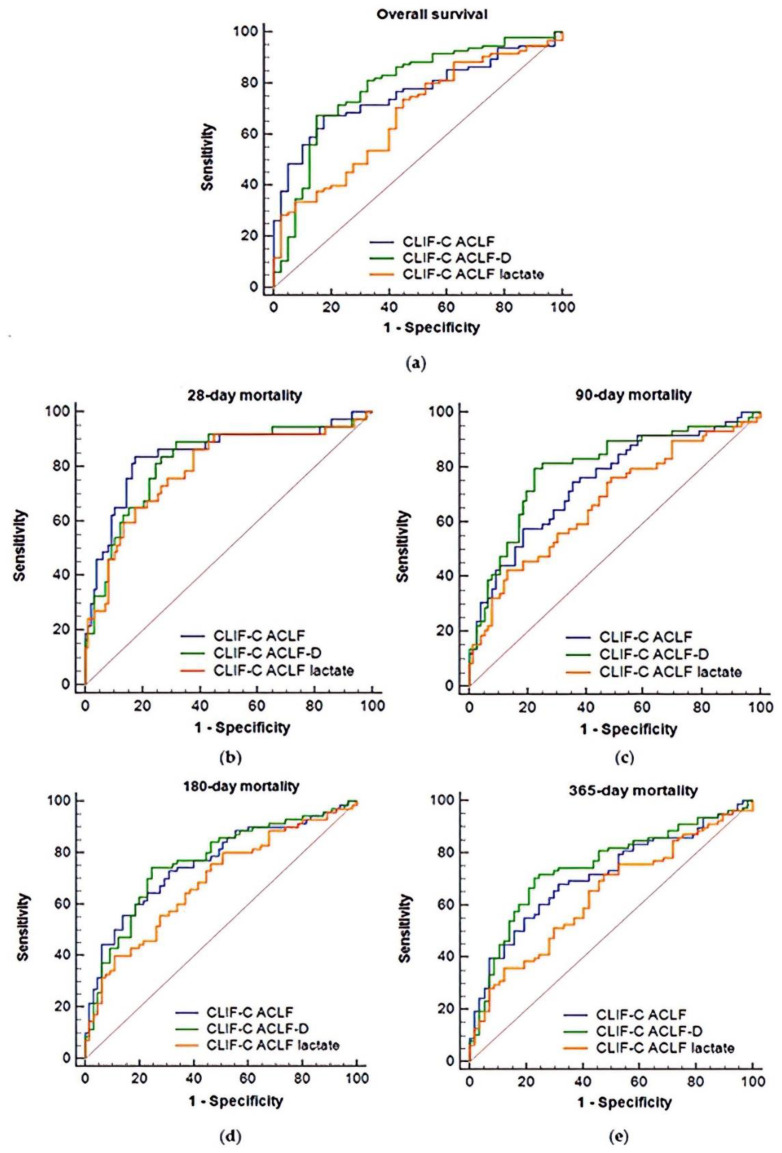
Comparison of the three prognostic scores predicting overall survival (**a**) and 28-day (**b**), 90-day (**c**), 180-day (**d**), and 365-day (**e**) mortality by AUROC analysis.

Furthermore, an ROC analysis of the three significant prognostic scores among 135 ACLF patients revealed AUROCs of the CLIF-C ACLF score (0.762 (0.682–0.841)) and CLIF-C ACLF-D score ((0.787 (0.699–0.875)) to be significantly higher than that of the CLIF-C ACLF_lactate_ score (0.673 (0.576–0.769)) for predicting overall mortality (Table 2 and Figure 3). Comparing overall (Figure 3a), day 28 (Figure 3b), day 90 (Figure 3c), day 180 (Figure 3d), and day 365 (Figure 3e) mortality prediction power by AUROC, the CLIF-C ACLF-D score displayed the highest AUROC, followed by the CLIF-C ACLF score and CLIF-C ACLF_lactate_ score. In addition, the CLIF-C ACLF and CLIF-C ACLF-D scores were statistically superior to the CLIF-C ACLF_lactate_ score in a pairwise comparison, although the three prognostic scores all showed good discriminatory performance (AUROC > 0.7).

## 4. Discussion

Previous studies have already demonstrated that the CLIF-C ACLF score provides a reliable and better alternative to other prognostic scoring systems for patients with ACLF [21]. However, a comparison of the CLIF-C ACLF score with two newer prognostic scores, the CLIF-C ACLF_lactate_ and CLIFACLF-D score, for patients hospitalized at our hepato-gastroenterologic ICU for the treatment of ACLF is intriguing and noteworthy. Overall, our data suggest that all three prognostic scores have sufficient prediction power for overall, short-term (28, 90-day), and long-term (180, 365-day) mortality among ACLF patients. The CLIF-C ACLF and CLIF-C ACLF-D scores were statistically superior to the CLIF-C ACLF_lactate_ in a pairwise comparison of AUROC. These scores may therefore be superior tools to evaluate patient prognosis in our ICU.

In order to reduce mortality in patients with ACLF, early liver transplantation plays a vital role given the short transplantation window [22,23]. This is especially true in East Asia, where HBV-ACLF is common [24]. Our results may help both hepatologists and intensivists more accurately predict prognosis in patients with ACLF. Prognosis is itself a critical factor in early identification of suitable liver transplant candidates.

A wide variety of prognostic scoring systems have been developed for assessing outcome and organ dysfunction among ICU patients [25,26]. However, low accuracy and weak prediction of short- and long-term mortality remain obstacles. Since the superiority of the CLIF-C ACLF score in prognostic prediction is known [27], further comparisons with two other newly developed scores were performed in the study. Although serum lactate level on admission to ICU seems useful to predict ICU mortality and a few calculated models have been discussed [10,28], severe hyperlactatemia does not have a completely positive correlation with ACLF mortality in our data. In our study, the correlation between lactate and 28-, 90-, 180-, 365-day and overall survival all revealed no correlation upon Pearson correlation analysis (*p* value = 0.787, 0.286, 0.406, 0.176, and 0.204, respectively). It is known that lactate level serves as a marker for tissue hypoxia and that the liver plays an important role in lactate clearance [29]. Although hepatic dysfunction is associated with higher serum lactate levels, hyperlactatemia is attributed to several causes, especially in critical patients with extremely high lactate level [30]. Several causes of hyperlactemia, such as cardiac failure, sepsis, or mesenteric ischemia, may confound the results. Moreover, reversible causes such as surgery or seizure can also influence the dynamic lactate level [31]. Close monitoring of serum lactate every 1–2 h has been recommended in acute patients but is often infeasible in clinical practice [31]. In fact, this is seldom performed at our clinic. Our limited lactate measurements may paradoxically explain why the CLIF-C ACLF and CLIF-C ACLF-D scores outperform for short- and long-term mortality prediction. Our results also confirm the recent PREDICT study finding that CLIF-C ACLF-D possesses high prognostic accuracy and is non-inferior to CLIF-C ACLF [10].

In our study, HBV-ACLF patients accounted for the majority of individuals studied. Male predominance among non-survivors was also noted, which may relate to the etiology of liver disease and different genotypes of hepatitis B virus infection between Europe and Taiwan [32]. The 28-day mortality in our study was lower (37/135 = 27.4%) than conventionally defined for ACLF. This may be explained by the demand for a specialized hepato-gastroenterologic ICU and ongoing universal hepatitis B vaccination for more than three decades [33].

There were limitations encountered in this study. First, as this study was done at a single large hepato-gastroenterologic academic center, referral bias may exist. However, the study continued given the demand for precise prediction of mortality in patients with ACLF [21]. Second, data obtained in the study were evaluated within 24 h after ICU admission. The CANONIC study obtained data between days 3 and 7 to calculate the CLIF-C ACLF score. Different clinical assessment time points for calculating the scores in patients with ACLF may lead to different results [28].

## 5. Conclusions

Our study demonstrates that the CLIF-C ACLF, CLIF-C ACLF-D, and CLIF-C ACLF_lactate_ scores are good and independent predictors of overall, and short- and long-term outcome among critically ill patients with liver cirrhosis and ACLF admitted to our ICU. The CLIF-C ACLF-D score may be a more powerful predictor of 28-, 90-, 180-, 365-day mortality and overall survival according to this single-center experience in Taiwan. Further prospective study is warranted to compare these scores for better prioritization of liver transplantation and improved prognostic accuracy in patients with cirrhosis and ACLF.

## Figures and Tables

**Table 1:** Baseline clinical parameters according to survival status after 28 days.

Parameter	All Patients (135 Patients)	Survivors (98 Patient)	Non-Survivors (37 Patients)	*p*-Value
**Male sex**	100 (66.66%)	79 (80.61%)	21 (56.75%)	0.02
**Age**	54.76 ± 14.06	54.21 ± 13.95	56.22 ± 14.46	0.50
**Etiology**				
HBV	54 (40.00%)	39 (39.80%)	15 (40.54%)	0.39
HCV	22 (16.29%)	12 (12.24%)	10 (27.02%)	0.04
ALC	35 (25.93%)	32 (32.65%)	3 (8.11%)	<0.01
HBV + HCV	6 (4.44%)	4 (4.09%)	2 (5.41%)	0.74
ALC + HCV	8 (5.93%)	5 (5.10%)	3 (8.11%)	0.51
Others	10 (7.41%)	6 (6.12%)	4 (10.81%)	0.33
**Laboratory values**				
Bilirubin(mg/dl) *	3.5 (1.7–9.1)	2.8 (1.68–4.88)	14.1 (4–26.5)	<0.01
INR *	1.6 (1.4–2.0)	1.5 (1.4–1.8)	2.1 (1.7–3.6)	<0.01
Albumin(g/dl) *	2.69 (2.40–3.01)	2.72 (2.41–3.04)	2.65 (2.21–2.85)	0.11
Sodium (mEq/L) *	138 (135–141)	138 (135–141)	140 (134–145)	0.16
Serum Cr (mg/dl) *	1.08 (0.79–1.73)	1.00 (0.77–1.28)	1.63 (1.16–2.26)	<0.01
WBC count (10^9^/L) *	8.7 (6–11.3)	8.1 (6–10.8)	10.4 (6.8–17.5)	0.01
Arterial PH *	7.42 (7.34–7.47)	7.43 (7.35–7.47)	7.41 (7.34–7.49)	0.94
HE grade I-II	56 (41.48%)	40 (40.81%)	16 (43.24%)	0.79
HE grade III-IV	17 (12.59%)	6 (6.12%)	11 (29.73%)	<0.01
**ACLF grades of EASL-CLIF consortium**				
ACLF1	68 (50.37%)	58 (59.18%)	10 (27.03%)	<0.01
ACLF2	22 (16.30%)	15 (15.31%)	5 (13.51%)	0.59
ACLF3	45 (33.33%)	25 (25.51%)	22 (59.46%)	<0.01

* Mean value presented by median and interquartile range.

**Table 2:** Area under the receiver operating characteristic curve (AUROC) of each score with pairwise comparison and different follow-up periods of mortality.

Score		AUROC (95%CI)	Pairwise Comparison of ROC Curves		
			A	B	C
**Overall survival**					
CLIF-C ACLF	A	0.762 (0.681–0.831)		*p* < 0.01	*p* = 0.53
CLIF-C ACLF_lactate_	B	0.673 (0.587–0.751)	*p* < 0.01		*p* = 0.01
CLIF-C ACLF-D	C	0.787 (0.708–0.853)	*p* = 0.53	*p* = 0.01	
**28-day mortality**					
CLIF-C ACLF	A	0.845 (0.773–0.902)		*p* = 0.03	*p* = 0.57
CLIF-C ACLF_lactate_	B	0.792 (0.713–0.857)	*p* = 0.03		*p* = 0.55
CLIF-C ACLF-D	C	0.821 (0.746–0.882)	*p* = 0.57	*p* = 0.55	
**90-day mortality**					
CLIF-C ACLF	A	0.747 (0.665–0.818)		*p* < 0.01	*p* = 0.23
CLIF-C ACLF_lactate_	B	0.673 (0.587–0.751)	*p* < 0.01		*p* < 0.01
CLIF-C ACLF-D	C	0.795 (0.717–0.860)	*p* = 0.23	*p* < 0.01	
**180-day mortality**					
CLIF-C ACLF	A	0.759 (0.678–0.828)		*p* < 0.01	*p* = 0.91
CLIF-C ACLF_lactate_	B	0.685 (0.600–0.763)	*p* < 0.01		*p* = 0.07
CLIF-C ACLF-D	C	0.763 (0.683–0.832)	*p* = 0.91	*p* = 0.07	
**365-day mortality**					
CLIF-C ACLF	A	0.711 (0.626–0.786)		*p* = 0.01	*p* = 0.07
CLIF-C ACLF_lactate_	B	0.636 (0.548–0.717)	*p* = 0.01		*p* = 0.01
CLIF-C ACLF-D	C	0.744 (0.662–0.815)	*p* = 0.07	*p* = 0.01	

AUROC: area under the receiver operating characteristic curve; the green blocks represent significant results by pairwise comparison.
